# Factors Affecting Subjective Well-Being Among Older Adults in India

**DOI:** 10.1093/geroni/igaa057.1093

**Published:** 2020-12-16

**Authors:** Kasturi Banerjee, Tamara Baker

**Affiliations:** University of Kansas, Lawrence, Kansas, United States

## Abstract

India has a rapidly expanding aging population whose unique mental health needs remain largely unexplored. Existent preliminary data however, show a significant association between life satisfaction and depressive symptoms within this population. Yet, little is known regarding the specific social and behavioral factors that may influence this relationship. Using data from the Longitudinal Aging Study in India (LASI) Pilot survey, the current study aimed to examine demographic and psychosocial factors associated with life satisfaction and subjective well-being among older adults 45+ years of age in the Indian states of Punjab, Rajasthan, Kerala and Karnataka. Results from the multivariate analyses indicated that age, household resources, neighborhood safety, religion, literacy status and participation in social activities are significantly associated with life satisfaction. Belonging to a southern state (β=.156; p<0.001), being financial provider status (β=-.073; p<0.001) and not being a care provider (β=.105; p<0.01) were significant predictors of greater life satisfaction. These findings are consistent with previous exploration of state level disparities regarding accessible resources and quality of life, and similarly the need to better understand the role of financial difficulties and care-giving burden among this population. These findings suggest the need to use qualitative assessment that explores the role additional factors such as social engagement and perceived neighborhood support have on this population’s subjective well-being; thereby shaping public policy, focus resources, and form the foundation of intervention programs.

